# Feature Extraction in Motor Activity Signal: Towards a Depression Episodes Detection in Unipolar and Bipolar Patients

**DOI:** 10.3390/diagnostics9010008

**Published:** 2019-01-10

**Authors:** Laura A. Zanella-Calzada, Carlos E. Galván-Tejada, Nubia M. Chávez-Lamas, M. del Carmen Gracia-Cortés, Rafael Magallanes-Quintanar, José M. Celaya-Padilla, Jorge I. Galván-Tejada, Hamurabi Gamboa-Rosales

**Affiliations:** 1 Unidad Académica de Ingeniería Eléctrica, Universidad Autónoma de Zacatecas, Jardín Juarez 147, Centro, Zacatecas 98000, Zac, Mexico; lzanellac@uaz.edu.mx (L.A.Z.-C.); tiquis@uaz.edu.mx (R.M.-Q.); gatejo@uaz.edu.mx (J.I.G.-T.); hamurabigr@uaz.edu.mx (H.G.-R.); 2 Unidad Académica de Odontología, Universidad Autónoma de Zacatecas, Jardín Juarez 147, Centro, Zacatecas 98000, Zac, Mexico; nubiachavez@uaz.edu.mx (N.M.C.-L.); gacc005340@uaz.edu.mx (M.d.C.G.-C.); 3 CONACYT—Universidad Autónoma de Zacatecas—Jardín Juarez 147, Centro, Zacatecas 98000, Zac, Mexico; jose.celaya@uaz.edu.mx

**Keywords:** depression, depresjon database, motor activity, feature extraction, classification, random forest

## Abstract

Depression is a mental disorder characterized by recurrent sadness and loss of interest in the enjoyment of the positive aspects of life, in addition to fatigue, causing inability to perform daily activities, which leads to a loss of quality of life. To monitor depression (unipolar and bipolar patients), traditional methods rely on reports from patients; nevertheless, bias is commonly present in them. To overcome this problem, Ecological Momentary Assessment (EMA) reports have been widely used, which include data of the behavior, feelings and other types of activities recorded almost in real time through the use of portable devices and smartphones containing motion sensors. In this work a methodology was proposed to detect depressive subjects from control subjects based in the data of their motor activity, recorded by a wearable device, obtained from the “Depresjon” database. From the motor activity signals, the extraction of statistical features was carried out to subsequently feed a random forest classifier. Results show a sensitivity value of 0.867, referring that those subjects with presence of depression have a degree of 86.7% of being correctly classified, while the specificity shows a value of 0.919, referring that those subjects with absence of depression have a degree of 91.9% of being classified with a correct response, using the motor activity signal provided from the wearable device. Based on these results, it is concluded that the motor activity allows distinguishing between the two classes, providing a preliminary and automated tool to specialists for the diagnosis of depression.

## 1. Introduction

The World Health Organization (WHO) defines that “health is a state of complete physical, mental and social well-being and not only of disease or infirmity”. More than 350 million people in the world suffer from depression and this can become a serious health problem, especially when it is of long duration and moderate to severe intensity, and can cause great suffering, disrupting work, school, family, economic and emotional activities, etc. In the worst case, it can lead to suicide, which is the cause of approximately 1 million deaths annually [1]. In Latin America, there is a high rate of mental health problems in the infant and youth population; about 20% of this population has disorders that require interventions of health services, but this number is underestimated due to the tendency of adolescents to hide and disguise their own problems to adults and their lack of confidence to access therapeutic structures [2]. Depression is a mental disorder characterized fundamentally by depressive mood, loss of interest and enjoyment of the positive aspects of life and fatigue, which impoverish the quality of life and generate difficulties in the family, work and social environment of those who suffer it [3].

Depression can manifest itself without taking into account age, sex, or socioeconomic status and can present with primary symptoms that do not include changes in mood and even do not change cognitive function, thus it is easy for any individual to fall into a depressive state [4]. Once depression has been developed, it is important that the subject undergoes a treatment that helps counteract the symptoms that this condition causes. However, one of the challenges that has arisen is that depression can be resistant to some treatments. Serafini et al. [5] studied the effects of repetitive transcranial magnetic stimulation (rTMS) in treatment-resistant depression, specifically on cognitive performance. According to the results, this noninvasive brain stimulation can be considered a technique for cognitive enhancement in depression that is resistant to treatments.

Traditional methods to monitor depression from unipolar and bipolar patient rely on reports from patients. However, in this type of monitoring, bias is commonly present. Additionally, changes in the behavior and understanding of the real world are present, as reported by Shiffman et al. [6]. An alternative to these reports is Ecological Momentary Assessment (EMA). EMA reports behavior, feelings and other type of activities as close as possible in time to the experience in real-life situations [6]. With the increasing of wearable devices (e.g., monitoring bands, smartwatches, etc.) and smartphones that include motion sensors, such as gyroscopes and accelerometers, EMA measurements can be done almost in real time, helping to monitor mental illness as well as given treatments and interventions, and increase the coverage of mental health services in the population without the need of new specific proposal devices.

For instance, smartphones and similar devices have been used in several approaches to tackle mental health illness. Gravenhorst et al. [7] discussed how mobile phones can support the treatment of mental disorders by two main approaches: implementing human–computer interfaces for therapy support and collecting relevant data from subjects’ daily lives to record the current state and the development of their mental problems, obtaining the advantages and drawbacks of the most promising technologies for detecting disorders such as depression or bipolar disorder. Firth et al. [8] demonstrated that psychological interventions using a smartphone as clinical media can reduce anxiety. Torous et al. [9] provided data on psychiatric patients and the relationship with the use and interest of utilizing mobile applications to monitor their mental health conditions. In this study, they claimed that 50% of patients from all age groups are interested in using a mobile application to control and monitor their mental health condition. Bayindir [10] presented a review of different works focused on mobile phone sensors used for the detection of human behavior characteristics, describing activity detection at different abstraction levels of activity and characterizing health-related activities, such as physical exercise and sleeping.

Devices which include sensors that can acquire contextual information are used to develop applications in several niches that include activity recognition [11,12,13]. Activity recognition can help to find mental disorders [14]. An approach proposed by Berle et al. [15] uses motor activity information to model the patterns of schizophrenia and depression disorders. Several apps have been proposed to offer self-help for depressed people. Nevertheless, Huguet et al. [16] concluded in their review that, even though these apps improve several aspects of cognitive behavioral therapy (CBT) or behavioral activation (BA) evaluation, utility of these are questionable, indicating the need for superior scientific, technological, and legal knowledge. Two interesting reviews were provided by Mohr et al. [17] and Guntuku et al. [18], who focused on mental health illness, using several sensing layers and sensor data to model behaviors and provide related mental health state. In addition to physical sensors, other approaches include data from social media, which include social networks (for instance, Twitter), online forums and public surveys. These approaches try to identify depressed states through the monitoring of these passive information of the activity of the subject. However, all these approaches need direct interaction of the patient, which can lead to errors in the final diagnosis. Thus, a method that minimizes the need of subject interaction to avoid “outliers”, who intentionally overfeed data, is required.

Therefore, the aim of this work was to obtain a method to detect depressive states through the motor activity of patients, using data from a smart-band, applying an approach of feature extraction from the activity developed by the patients, which will allow diagnosis and timely treatment. In this context, it is of great importance to expand the knowledge of the variables involved in the initiation and maintenance of anxious-depressive symptomatology in emotional disorders, such as those proposed in this research, based on the information of the physical activity of the patients, taking into account that depressive subjects are more likely to have less motor movement than controls. Deepening in these aspects can contribute to the prevention of this psychopathology, as well as to the development of effective treatments.

The hypothesis proposed refers to a method based on motor activity collected by a smart-band that allows the early diagnosis of depression and consequently a timely treatment, through the application of artificial intelligence techniques.

This paper is organized as follows. Section 2 presents a description of the data used for this research and the methods that were carried out. Results obtained from the feature extraction process and the validation step are shown in Section 3, as well as the the discussion of the behavior of the features extracted from the motor activity signal. Finally, conclusions are described in Section 4 and future work is presented in Section 5.

## 2. Materials and Methods

**Figure 1:**
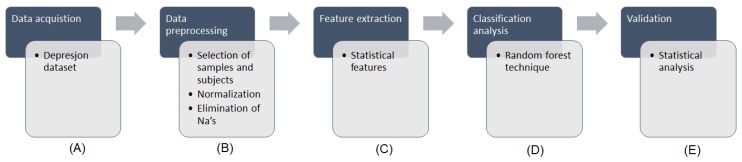
Flowchart of the methodology followed. Blue squares refers to the data processing step and gray squares details task dones in each step (**A**–**E**).

The methodology followed in this work is presented in Figure 1. Figure 1A shows the data that were obtained from the Depresjon database. Figure 1B presents the data preprocessing stage, consisting in the selection of samples and subjects from the original dataset, the normalization of data and the elimination of incomplete cases. The feature extraction, as shown in Figure 1C, was performed in order to extract a series of statistical features, which were subsequently subjected to a classification analysis (Figure 1D) through a random forest (RF) technique. Finally, a validation step as performed to evaluate the results obtained by measuring the receiver operating characteristic (ROC) curve and its area under the curve (AUC) correspondent value (Figure 1E).

### 2.1. Data Description

The Depresjon dataset contains information of patients with absence of depression (controls) vs. patients with presence of depression (cases). In this dataset, the activity levels were monitored through an actigraph watch worn on the right wrist. The actigraph watch used was the “Actiwatch” (Cambridge Neurotechnology Ltd., Cambridge, UK, model AW4), which has a sampling frequency of 32 Hz and records movements over 0.05 g. Movements are stored in the memory unit of the watch based on the corresponding voltage that is produced, thus the number of counts is proportional to the intensity of the movements. The total activity counts were recorded in intervals of one minute.

The features collected for each subject were divided in two categories: actigraph data recorded over time and Montgomery–Åsberg Depression Rating Scale (MADRS) scores. For this work, only the features over time were used, which include: timestamp (one minute intervals), date (date of measurement) and activity (activity measurement from the actigraph watch).

The Depresjon data used to support the findings of this study have been deposited in the “control” and “condition” repositories. This dataset can be accessed at http://datasets.simula.no/depresjon/ or directly downloaded from http://doi.org/10.5281/zenodo.1219550.

**Figure 2:**
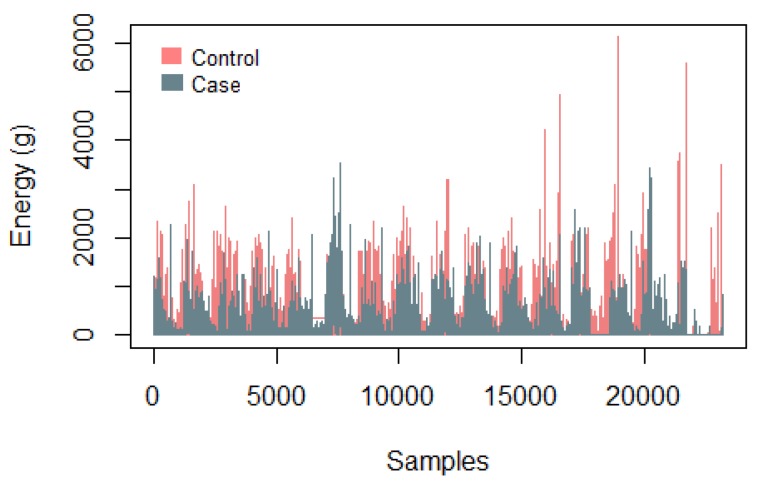
Example of samples collected with the Actiwatch of a control and a case of the Depresjon database.

Figure 2 presents a graph with the samples collected using the Actiwatch from a control subject and from a case subject to identify the energy level over time, where the difference in energy presented by each subject is evident, with the control subject presenting higher levels.

The total subjects contained in the Depresjon data set were 5895 (2112 cases/3783 controls).

### 2.2. Data Preprocessing

The data preprocessing consisted of three main steps: the selection of samples and subjects, the elimination of incomplete cases and the normalization of data.

Is is important to mention that these preprocessing steps were used to collect a balanced volume of data to carry out the proposed methodology, as well as to make the data present a standard distribution, that is, with an average of zero and a standard deviation of one. Besides, the elimination of incomplete cases allowed avoiding bias problems and reducing the value of uncertainty due to missing values.

The number of samples collected in the original datasets is not consistent, differing in the number of minutes recorded for each subject, thus a selection of subjects and samples was made to present a balanced volume of data referring to controls and cases. In the selection of the samples, only the first value of the 60 values acquired during 1 h was kept, equivalent to the minutes correspondent to that time lapse, counting now the activity in intervals of 1 h. This procedure was performed for each hour of the total data. On the other hand, the selection of subjects depended on the volume of data resulting from the selection of samples, looking for the most balanced volume of data possible,. The first four controls present in the dataset and the first five cases were selected, thus balancing the number of samples.

The elimination of incomplete cases consisted in removing all rows where any missing value was found, represented as NA (not available).

Then, the normalization was calculated with Equation (1), where $z_{i}$ represents the current value normalized, $x_{i}$ represents the original value, μ represents the mean of the column where the value is located and σ represents the standard deviation. This step was performed to avoid overfitting problems.

(1) $$z_{i}=\frac{x_{i}-\mu }{\sigma }$$

### 2.3. Feature Extraction

The feature extraction was performed to obtain a series of 14 statistical parameters, as presented in Table 1, which were subsequently analyzed. These features were extracted from the time dependant features of the database, which were collected from the activity of the subjects through the actigraph watch.

These statistical features were chosen because they are the first, second, third and fourth moments of an aleatory variable, which represent the descriptive measures that may be used for the characterization of the probability distribution of that variable. In other words, they describe the characteristics of the time courses of the activity measured [19].

### 2.4. Classification Analysis

In the classification analysis, the machine learning technique Random Forest (RF) was used for the classification of subjects in two different states: depressed (labeled as “1”) or not depressed (labeled as “0”).

RF is a non-parametric statistical method introduced by Breiman et al. [20], which has been widely used in different health approaches, such as the in development of models to identify high-risk surgical patients through the electronic health record data [21], the definition of the individual double minimum-distance of protein–RNA for the structure-based prediction [22], the prediction of plant-derived xenomiRs from plant miRNA sequences [23], the modeling of the groundwater nitrate exposure in private wells for the Agricultural Health Study [24], the classification of neuroimaging data in Alzheimer’s disease [25], and the development of a three-level hepatotoxicity prediction system based on adverse hepatic effects [26], among others.

On the other hand, a comparison between RF and other machine learning techniques has shown the benefits that RF presents over them. In the research of Muhammad et al. [27], the behavior of RF and artificial neural networks (ANN) is evaluated and compared. According to the results obtained, the RF technique can effectively handle missing values during both training and testing stages. Besides, the training time of RF is lower than that of ANN.

In addition, Brokamp et al. [28] presented a comparison between linear regression and RF approaches, identifying that regression models obtained lower accuracy when predicting certain values, which implies that the misclassification is differential and could cause biased associations with control outcomes, while RF models presented an increased accuracy and a decreased variance of prediction error.

Chen et al. performed a comparative study of logistic model tree, RF, and classification and regression tree models, finding that, even when the three techniques exhibited good performances, RF model obtained the highest predictive value [29].

In the study of Ahmadi et al. [30], logistic regression and RF techniques are compared, obtaining that RF outperforms logistic regression, according to the AUC, total accuracy and Kappa coefficient, showing that RF improves the prediction searched, concluding that this happened due to the former accounts for all interactions between covariates.

This technique is basically a set of decision trees, but improved, and it has three main approaches: classification, regression and unsupervised learning [31]. In the classification case, RF provides estimators of a Bayes classifier, f:R↦y, minimizing the error classification P(Y)≠f(X).

The decision trees of the RF are constructed over $n_{tree}$ bootstrap samples $L^{1},\ldots ,L^{n_{tree}}$ of a training set *L*, and it is based in a replacement principle, which is a bagging approach, meaning that the same sample can be used several times for the training stage, while others may be not selected [32].

In every node of the trees, a number of features, $m_{feature}$, is randomly selected to determine the splitting rule among them. This learning rule is added to the estimators resulting from the trees, represented as ${\hat{f}}_{1},\ldots ,{\hat{f}}_{n_{tree}}$. Then, a response value is obtained from the new point, consisting in building Equation (2).

(2) $$\hat{f}\left(x\right)=argmax1\leq c\leq C\sum_{k=1}^{n_{tree}}1_{{\hat{f}}_{k}\left(x\right)=c}$$

By growing the forest up to $n_{tree}$, the algorithm produces trees that have high variance and low bias. The final classification decision is taken by calculating the arithmetic mean of the class assignment probabilities obtained by all produced tress. Subsequently, new unlabeled data input are evaluated through all decision trees of the ensemble and each tree provides a vote for a class. The class with more votes is finally selected.

From the total amount of samples, about two thirds, called the in-bag samples, are the *L* selected to train the trees and the remaining one third, called the out-of-bag (OOB) samples, are used for an internal cross-validation to measure the performance of the RF model.

This value is known as the OOB error and is measured by the misclassification rate for the classification of the OOB samples. That is, a feature $X^{j}$ will be important if, when breaking the relationship between $X^{j}$ and *Y*, the prediction error increases, and the prediction error of each tree $\hat{f}$ is evaluated trough the OOB sample with Equation (3) [33].

(3) $$\hat{R}\left(\hat{f},\hat{L}\right)=\frac{1}{\left|L\right|}\sum_{i:\left(X_{i},Y_{i}\right)\epsilon \bar{L}}1_{\hat{f}\left(X_{i}\right)\neq Y_{i}}$$

For this work, the number of trees selected was $n_{tree}$ = 2000, and the number of features tried at each split was $m_{feature}$ = 3.

### 2.5. Validation

The evaluation of the results obtained was carried out in the validation stage through a ROC curve-based approach. The ROC curve has been widely used to measure or visualize a classifier’s performance in conjunction with the AUC value to select a suitable operating point, called as decision threshold [34].

The two possible outputs in a classification problem are “correct” and “incorrect” for each class. This information can be represented in a confusion matrix, which is a table that shows the differences between the true and predicted classes for a set of labeled examples. This table mainly contains the true positives (Tp), true negatives (Tn), false positives (Fp) and false negatives (Fn) values, as well as the row totals with the truly negatives (Cn) and truly positives (Cp) examples, and the column totals with the predicted negative (Rn) and the predictive positive (Rp) examples. From these parameters, more meaningful measures can be extracted to have certain performance criteria, such as the accuracy, which refers to the degree to which the result of a calculation conforms to the correct value, shown in Equation (4),

(4) $$accuracy\left(1-error\right)=\frac{Tp+Tn}{Cp+Cn};$$

the sensitivity, which is referred to the ability to correctly identify those with a condition, shown in Equation (5),

(5) $$sensitivity\left(1-\beta \right)=\frac{Tp}{Cp};$$

the specificity, which is referred to the ability to correctly identify those without a condition, shown in Equation (6),

(6) $$specificity\left(1-\alpha \right)=\frac{Tn}{Cn};$$

the positive predicted value (PPV), which is the proportion of true positives results, shown in Equation (7),

(7) $$PPV=\frac{Tp}{Rp};$$

and the negative predicted value (NPV), which is the proportion of true negatives results, shown in Equation (8).

(8) $$NPV=\frac{Tn}{Rn}.$$

The plotted values of the sensitivity and the specificity as the decision threshold is called ROC curve, and the simplest way to calculate the AUC is through trapezoidal integration, shown in Equation (9),

(9) $$AUC=\sum_{i}\left(1-\beta_{i}\cdot \Delta \alpha \right)+\frac{1}{2}\left[\Delta \left(1-\beta \right)\cdot \Delta \alpha \right]$$

where $\Delta \left(1-\beta \right)=\left(1-\beta_{i}\right)-\left(1+\beta_{i-1}\right)$ and $\Delta \alpha =\alpha_{i}+\alpha_{i-1}$.

All analyses performed in this work were carried on in “R” (version 3.4.4) [35], which is a “free software environment for statistical computing and graphics”. The libraries used were “randomForest” (version 4.6-14) [36], “e1071” (version 1.7-0) [37], “pROC” (version 1.11.0) [38], “caret” (version 6.0-79) [39] and “rminer” (version 1.4.2) [40].

## 3. Results and Discussion

In this section, the results obtained for each stage of the methodology are exposed.

Initially, from the data preprocessing step, the total subjects were reduced to 4125 (controls = 2176/cases = 1949) and the total data were standardized, causing them to have a mean = 0 and a standard deviation = 1.

According to these results, the number of data selected from the total dataset was adequate to be able to carry out the proposed methodology obtaining significant results, even when the data were slightly unbalanced, because the volume of data originally acquired was presented in greater quantity for controls than for cases.

Then, a set of 14 statistical features were extracted based in the statistical moments to know the performance of the data through the time, looking for alterations at specific times that could provide meaningful information.

Then, from the classification analysis through the RF technique, an OOB estimate of error rate of 8.95% was obtained, which implies that this percentage of the OOB samples were incorrectly classified through the internal cross-validation of RF.

It is worth mentioning that some of the main reasons for choosing this technique for the classification analysis were that it can be used for handling high-dimensional data, it performs an internal cross-validation, it only has a few tuning parameters, it is easy to interpret even when the relationships between predictors are complex, it uses all available input variables simultaneously, and it has an intuitive structure. Besides, as it is a non-parametric method, it is not necessary to comply with any specific distribution, thus it requires less preprocessing of data compared to other statistical learning methods and it is not greatly influenced by outliers [27].

Then, the sensitivity, specificity and error rates were also calculated, as shown in the confusion matrix of Table 2 (where the top indices represent the predicted values, the lateral indices represent the reference values and the last column presents the error rate for the specificity and sensitivity, respectively), to measure the performance of the learning stage of the algorithm, where it is possible to observe that, from the in-bag samples, which correspond to controls = 1483/cases = 1267, the error rate of the specificity is 0.077, meaning that at least 92.3% of the controls were correctly classified, and the error rate of the sensitivity is 0.104, meaning that at least 89.6% of the cases were correctly classified.

These values represent statistically significant results, since a low percentage of subjects was misclassified in the learning process of RF, which implies that the information contained in the extracted features is presenting values that allow distinguishing between the two possible classes of the subjects. This discussion is supported with the OOB error, validating through a test the performance of the learned model.

**Figure 3:**
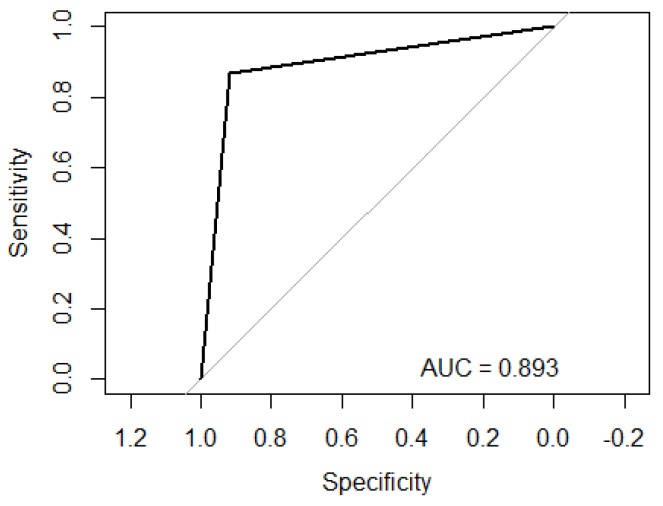
ROC curve obtained from the classification analysis based in RF.

On the other hand, from the validation stage, Figure 3 is obtained, which shows the ROC curve of the performance of modeling the data through RF, obtaining an AUC of 0.893. As can be seen, the AUC value matches with the results obtained from the internal validation of RF; therefore, it also presents a significant specificity/sensitivity rate.

Table 3 presents a confusion matrix based on a blind test, using 30% of the data (unlabeled) to evaluate the performance of the classification, where the top indices represent the reference values and the lateral indices represent the predicted values. For this test, the subjects were randomly selected and balanced, using a total of 1375 (controls = 693/cases = 682).

These results show true positives of 591 and true negatives of 637 (false positives of 56/false negatives of 91), also being statistically significant and supporting the results obtained previously.

Finally, Table 4 presents a set of measures calculated to validate the ability to classify subjects based in RF, allowing to complement and understand the significance of the performance criteria. The accuracy shows a value of 0.893, which means that, for any subject, there is a degree of 89.3% of being classified with a correct value. Then, the sensitivity shows a value of 0.867, referring that those subjects with presence of depression have a degree of 86.7% of being correctly classified. The specificity shows a value of 0.919, referring that those subjects with absence of depression have a degree of 91.9% of being classified with a correct response.

It is important to remark that, in all results, the classification rate of controls presents better values than the classification rate of cases, which may be because in some time ranges the subjects with presence of depression can be presenting similar information to subjects with absence. Frequently, depressive patients have less activity in their daily life. This could cause confusion when a non-depressed patient shows low activity in a specific time range, for example at night while sleeping, inflicting on the depressive patient being classified as non-depressive.

The balanced accuracy was calculated to support the value of the regular accuracy, because, even when the data were not significantly unbalanced, the number of controls exceeded the number of cases. This parameter obtained a value of 0.892, which is only 0.1% lower than the regular accuracy, validating the statistically significant result calculated previously.

The PPV and NPV, which obtained values of 0.875 and 0.931, respectively, were calculated to know the proportion of true positives and the proportion of true negatives, where the results agree with the other validation parameters and it is corroborated that the subjects with absence of depression were slightly better classified than the subjects with presence.

Finally, the results outperform the baseline performance proposed by García-Ceja et al. [41], which includes Nearest Neighbors, Linear kernel Support Vector Machine (SVM), Radial Basis Function kernel (RBF) SVM, Gaussian Process, Decision Tree, Random Forest, Neural Network, AdaBoost, Naive Bayes, and Quadratic Discriminant Analysis (QDA), as presented in Table 5.

## 4. Conclusions

This study proposed an analysis to find the relationship between a series of statistical features, based on continuous values acquired in a specific time, and the possible condition of depression.

Since the number of patients was adequate to carry out this research and the extracted features allowed describing the main characteristics of a patient’s full-day activity, according to the modeling developed between them and the condition of presence or absence of depression, evaluated through statistical validation, it is possible to conclude that the results obtained through this methodology shown statistically significant values indicating that there is an association between the recorded daily activity of a patient and the condition of his depressive state.

Among the symptoms presented by patients with depression are the slowness of movement, poor body gesticulation and the feeling of fatigue, thus they tend to show lower levels of activity than subjects who do not have this condition, giving meaning to the results obtained.

Therefore, the main benefit presented in this study is a preliminary tool (bearing in mind that it is necessary to study in greater depth this approach, taking into account the regulations of the health system and characterizing the results) that may support the diagnose of specialists to know if a patient presents depression based on the level of activity he has in a full day through the automatic diagnosis of subjects obtained by submitting this information to the model developed in this work, relating the total motor activity with the presence or absence of depression, which is shown, according to the results presented, to have a significantly high accuracy, allowing to reduce false positives and false negatives in the detection of this condition, thus improving the diagnosis of this disease..

Through this research is obtained a second automatic opinion of low cost, since the implementation of the developed model does not need any software or specialized hardware, so it is viable to be used in regions with limited access to health services.

Finally, it is important to mention that the main limitation of this study is the large volume of data on the motor activity required by each patient, which makes the analysis of the data a bit complex and delayed, causing a small number of subjects to be used. On the other hand, a limiting factor that could broaden the focus of this work would be to know information about patients in relation to any psychiatric or other treatment (e.g., sleeping pills) that they are currently undergoing, allowing us to know if this influences the amount of activity performed by the subjects, which could affect the controls by reducing their physical activity, causing false positives.

## 5. Future Work

As future work, it is proposed to increase the number of subjects in the experimentation to present results based on a greater diversity of data and thus highlight the robustness of the results. In addition, the inclusion of a stage of feature selection is proposed, comparing different machine learning tools, to know which are the features that have the greatest contribution in the classification of depressive and non-depressed subjects.

References

## Figures and Tables

**Table 1:** Statistical features collected.

Feature	Description
Mean	$\mu =1/n\sum_{i=1}^{n}x_{i}$
Standard deviation	$sd=\sqrt{\sum_{i=1}^{N}{\left(x_{i}-\mu \right)}^{2}/\left(N-1\right)}$
Variance	$sd^{2}=1/n\sum_{i=1}^{n}{\left(x_{i}-\mu \right)}^{2}$
Trimmed mean	Mean with outliers trimmed.
Coefficient of variation	CV=sd/μ
Inverse coefficient of variation	ICV=μ/sd
Kurtosis	K=μ/σ
Skewness *	S=(μ−υ)/σ
Quantile * 1, 5, 25, 75, 95, 99%	Q[i](p)=(1−γx[j]+γx[j+1]

* υ represents the median value; 1 ≤ *i* ≤ 9, (j−m)/n≤ *p* <(j−m+1)/n; x[j] represents the *j*th order statistic; *n* represents the sample size; γ is in function of *j* and *g*, where j=floor(np+m) and g=np+m−j; and *m* represents a constant determined by the sample quantile type.

**Table 2:** Confusion matrix of the subjects classification based in the RF approach.

	Control	Case	Error
**Control**	1369	114	0.077
**Case**	132	1135	0.104

**Table 3:** Confusion matrix of the validation through a blind test.

	Control	Case
**Control**	637	91
**Case**	56	591

**Table 4:** Parameters obtained through validation.

Parameter	Value
Accuracy	0.893
Sensitivity	0.867
Specificity	0.919
Balanced accuracy	0.892
PPV	0.875
NPV	0.931

**Table 5:** Machine learning techniques comparison.

Technique	Specificity	Accuracy
Nearest Neighbors	0.696	0.675
Linear SVM	0.726	0.727
Random Forest	0.703	0.700
Neural Net	0.716	0.719
AdaBoost	0.707	0.706
Naive Bayes	0.688	0.694
Our proposal (Feature extraction & RF)	0.919	0.893
